# Effect of sodium cantharidinate/vitamin B6 injection on survival, liver function, immune function, and quality of life in patients with hepatocellular carcinoma: Protocol for a meta-analysis

**DOI:** 10.1097/MD.0000000000021952

**Published:** 2020-08-21

**Authors:** Min Zhu, Xiujing Liu, Changhui Zhou, Juan Li

**Affiliations:** a Department of Clinical Laboratory; b Department of Central Laboratory, Liaocheng People's Hospital, Liaocheng, Shandong Province, P.R. China.

**Keywords:** efficacy, hepatocellular carcinoma, immune function, liver function, quality of life, sodium cantharidinate/vitamin B6

## Abstract

**Background:**

Sodium cantharidinate/vitamin B6 (SC/VB6) injection, a famous insect-derived traditional Chinese medicine preparation, has been widely applied as a promising adjunctive drug for hepatocellular carcinoma (HCC). However, its exact clinical efficacy and safety is still not well investigated. In this study, we aimed to summarize the efficacy of SC/VB6 injection on survival, liver function, immune function, and quality of life (QoL) in patients with HCC through the meta-analysis.

**Methods:**

All available randomized controlled trials (RCTs) and high-quality prospective cohort studies that investigated the efficacy and safety of SC/VB6 for patients with HCC were searched from ten electronic databases including PubMed, Google Scholar, Cochrane Library, Excerpt Medica Database (Embase), Medline, Web of Science (WOS), Chinese Biomedical Literature Database (CBM), China National Knowledge Infrastructure (CNKI), China Scientific Journal Database (CSJ), and Wanfang Database. Papers in Chinese or English published from January 2000 to July 2020 will be included without any restrictions.

Study selection and data extraction will be performed independently by 2 researchers. The clinical outcomes including overall survival (OS), QoL, liver function, immune function, and adverse events, were systematically evaluated. Review Manager 5.3 and Stata 14.0 were used for data analysis, and the quality of the clinical trials was also evaluated.

**Results:**

The results of this study will be published in a peer-reviewed journal, and provide a helpful evidence for clinicians to formulate the best postoperative adjuvant treatment strategy for HCC patients.

**Conclusion:**

Our study will draw an objective conclusion of the efficacy of SC/VB6 on survival, liver function, immune function, and QoL in patients with HCC.

**INPLASY registration number:**

INPLASY202070121.

## 1 Introduction

Hepatocellular carcinoma (HCC) is the seventh most commonly diagnosed malignancy and the second most frequent cause of tumor-related death.^[1,2]^ According to statistics, about 841,080 newly diagnosed cases and 781,631 deaths occurred worldwide in 2018.^[1,2]^ Among them, about 50% of newly diagnosed patients were occurred in China.^[3]^ The main factors that cause HCC are hepatitis B virus (HBV) and hepatitis C virus (HCV) infection, excessive alcohol consumption, contact or consumption of Aspergillus toxins as well as various metabolic disorders.^[4,5]^ Despite the improvement of diagnostic and treatment methods in recent years, the prognosis of HCC remains unsatisfactory.^[6–8]^ More than half HCC patients already have advanced or metastatic lesions when diagnosed, due to the lack of noticeable clinical symptoms, and the 5-year survival rate of advanced HCC patients was <20%.^[9,10]^ Currently, the clinical treatment of HCC mainly includes radiotherapy, chemotherapy, surgical resection alone or combined strategy.^[9,10]^ However, it is known that the above conventional therapeutic methods often fail to remove the tumor completely.^[9,11]^ In addition, the unpleasant side effects related to chemoradiotherapy also seriously affects the quality of life (QoL) and immune function of patients with HCC.^[9,11]^ Therefore, exploring new alternative regimens with better tolerance and lower toxicity for HCC patients are urgently required.

Traditional Chinese medicine has been used as an complementary treatment for alleviating the side effects of radiochemotherapy, improving the QoL and immune function of cancer patients.^[12–14]^ Many scholars pointed out that the combination of Chinese and Western medicine for HCC may be the potential trend of clinical treatment in the future.^[12–14]^ Sodium cantharidinate/vitamin B6 (SC/VB6, a famous insect-derived traditional Chinese medicine) is a combination of sodium cantharidinate and VB6, and has the pharmacological characteristics of both.^[11,15,16]^ SC is a derivative of cantharidin, which is extracted from the body of meloidae insects such as *Mylabris phalerata pallas* and *Mylabris cichorii linnaeus*.^[11,17]^ SC preserves the unique anti-cancer activity of cantharidin and has lower toxicity and fewer adverse effects.^[11,15,17]^ Its combination with VB6 can even further lower the side effects.^[11,15]^ Previous studies suggest that the anti-tumor mechanisms of SC might be attributed to the following aspects:

1. SC can significantly inhibit tumor cell proliferation and induces cell cycle arrest by inhibition of PI3K/AKT activation^[18,19]^;
2. SC can induces HepG2 cells apoptosis through the LC3 autophagy and mitochondrial pathway^[20]^;
3. SC can exert the anti-tumor efficiency through depressing the expression of vascular endothelial growth factor and blocking tumor angiogenesis.^[21]^

Other studies suggest that SC also can effectively increase the sensitivity of tumor cells to chemotherapeutic agents.^[11,22]^

Several studies have indicated that the combination of SC/VB6 and classic radiochemotherapy not only exerts an enhanced therapeutic effect against HCC, but also improve the QoL and immune function of patients.^[11,16,22]^ Despite the intensive clinical studies, its clinical efficacy was still not well investigated. In this study, we are prepared to summarize the efficacy of SC/VB6 on survival, liver function, immune function, and QoL in patients with advanced HCC through the meta-analysis, in order to provide a helpful evidence for clinicians to formulate the best postoperative adjuvant treatment strategy for HCC patients.

## 2 Review question

Is SC/VB6 injection effective on survival, liver function, immune function, and QoL in patients with HCC?

## 3 Objective

A systematic review and meta-analysis will be performed to systematically evaluate the efficacy of SC/VB6 injection on survival, liver function, immune function, and QoL in patients with HCC.

## 4 Methods

The protocol of this meta-analysis will be reported according to Preferred Reporting Items for Systematic Review and Meta-Analysis Protocols (PRISMA-P) guidelines.^[23]^ Our protocol has been registered on the International Platform of Registered Systematic Review and Meta-Analysis Protocols (INPLASY). The registration number was INPLASY202070121 (DOI number is 10.37766/inplasy2020.7.0121, https://inplasy.com/inplasy-2020-7-0121/). This meta-analysis is a secondary research which based on some previously published data. Therefore, the ethical approval or informed consent was not required.

### 4.1 Search strategy

To perform a comprehensive and focused search, experienced systematic review researchers will be invited to develop a search strategy. The plan searched terms are as follows: “sodium cantharidinate” or “disodium cantharidinate” or “vitamin B6” or “sodium cantharidinate/vitamin B6” or “disodium cantharidinate/vitamin B6” or “aiyishu” or “banmaosuanna weishengsuB6 zhusheye” or “SC/VB6” or “DC/VB6” combined with “liver cancer” or “liver carcinoma” or “hepatocellular cancer” or “hepatocellular carcinoma” or “LC” or “HC” or “HCC” et al. An example of search strategy for PubMed database shown in Table 1 will be modified and used for the other databases.

**Table 1:**
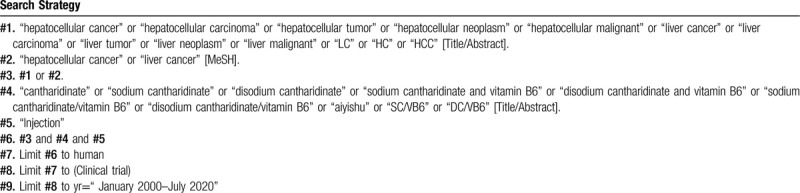
Searching strategy in PubMed.

### 4.2 Eligibility criteria

#### 4.2.1 Types of studies

All available randomized controlled trials (RCTs) or quasi-RCTs, and high-quality prospective cohort studies that investigated the efficacy of SC/VB6 injection on survival, liver function, immune function, and QoL in patients diagnosed with advanced HCC will be included in this systematic review.

#### 4.2.2 Types of participants

Patients must be cytologically or pathologically confirmed as having HCC at a clinically advanced stage. No restrictions regarding age, gender, racial, region, education, and economic status in this analysis. Patients with other malignancies or non-primary HCC are not included.

#### 4.2.3 Types of interventions

HCC patients in the experimental group must be treated with conventional treatment (including radiotherapy, chemotherapy, and targeted therapy) combined with SC/VB6 injection.

#### 4.2.4 Comparator

In the control group, HCC patient treated with the same conventional treatment as experimental group.

#### 4.2.5 Exclusion criteria

Papers without sufficient available data, non-comparative clinical trials, non-peer reviewed studies, meta-analysis, literature reviews, case reports, meeting abstracts, letter to the editor, commentaries, and other unrelated researches will be excluded from analysis.

### 4.3 Information sources

Electronic databases including PubMed, Google Scholar, Cochrane Library, Excerpt Medica Database (Embase), Medline, Web of Science (WOS), Chinese Biomedical Literature Database (CBM), China National Knowledge Infrastructure (CNKI), China Scientific Journal Database (CSJ), and Wanfang Database will be systematically searched for eligible clinical trials from January 2000 to July 2020. Language is limited with English and Chinese.

### 4.4 Types of outcome measures

#### 4.4.1 Primary outcomes

- Overall survival (OS, which is defined as the time from the date of randomization to death from any cause);
- Immune function indicators: CD3^+^, CD4^+^, CD8^+^, NK cells percentage, and CD4+/CD8+ cell ratios, and serum cytokines level (IL-2, IL-4, IFN-γ, and TNF-α);
- Liver function assessment: Liver function of patients with HCC was assessed in terms of total bilirubin (TBIL), serum albumin (ALB), alanine aminotransferase (ALT), and aspartate aminotransferase (AST) levels, prothrombin time (PT), and prothrombin activity (PTA);
- QoL as evaluated by Karnofsky score.

#### 4.4.2 Secondary outcomes

Secondary outcomes will include:

- Overall response rate (ORR, complete response + partial response) and disease control rate (DCR, complete response + partial response + stable disease);
- Alpha-fetoprotein (AFP) level;
- Adverse events: toxicity was graded from 0 to IV in severity on the basis of the World Health Organization (WHO) recommendations.

### 4.5 Data collection and analysis

We will adopt the measures described in the Cochrane Handbook for Systematic Reviews of Interventions to pool the evidence.^[24]^

#### 4.5.1 Study selection and management

**Figure 1:**
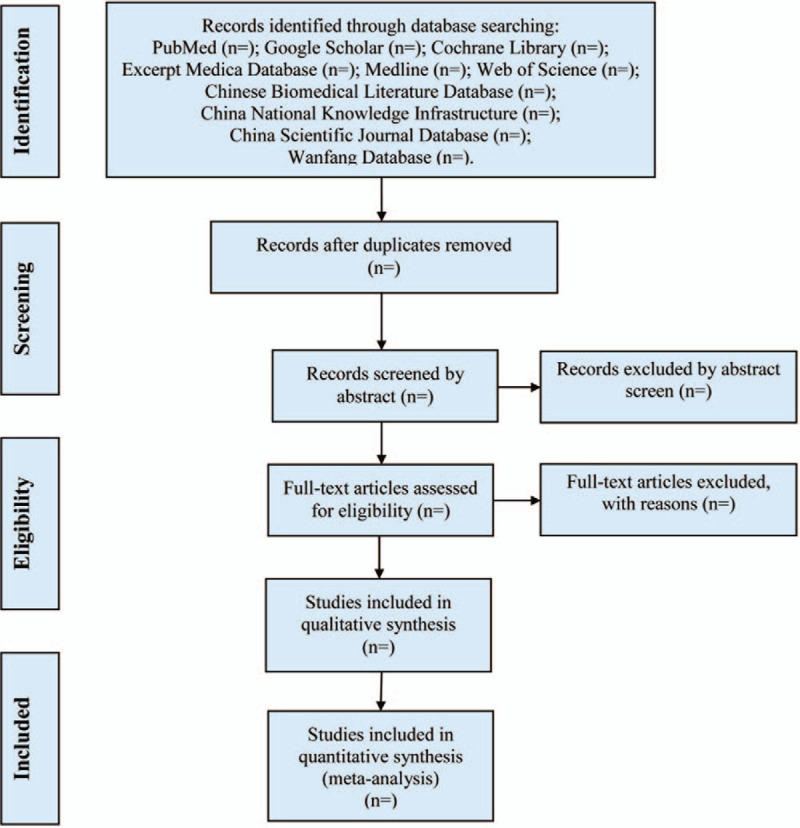
Study selection process for the meta-analysis.

Two authors (Zhu M and Liu XJ) will be reviewed independently to identify potential trials by assessing the titles and abstracts and identify whether the trials meet the inclusion criteria. The full text will be further reviewed to exclude irrelevant studies or determine potential eligible studies. Endnote X7 software will be used for literature managing and records searching. Disagreements between the two reviewers will be resolved by discussing with the third investigator (Zhou CH). Excluded studies and the reasons for exclusion will be listed in a table. A PRISMA-compliant flow chart (Fig. 1) will be used to describe the selection process of eligible literatures.

#### 4.5.2 Data extraction and management

Two investigators (Zhu M and Liu XJ) will be responsible for the data extraction independently according to the Cochrane Handbook for Systematic Reviews of Intervention.

The following data will be extracted from eligible literatures:

**Study characteristics and methodology:** country of study, the first author, year of publication, study design, sample size, periods of data collection, total duration of study, and follow-up duration, et al.
**Participant characteristics:** tumor stage (staging of the tumor according to the AJCC TNM classification for esophageal cancer), age, gender, ethnicity, pathology diagnosis, pathologic tumor size, inclusion and exclusion criteria, et al.
**Interventions:** therapeutic means, manufacturer of the drugs, dosage of SC/VB6, administration route and cycles, duration of treatment and follow-up time, et al.
**Outcome and other data:** ORR, OS, QoL, immune indexes (CD3^+^, CD4^+^, CD8^+^, NK cells percentage, and CD4+/CD8+ cell ratios, and serum cytokines level [IL-2, IL-4, IFN-γ, and TNF-α]), Liver function indicators (TBIL, ALB, ALT, AST, PT, and PTA), AFP and adverse effects, et al.
**Dealing with missing data:** we will attempt to contact the authors to request the missing or incomplete data. If those relevant data are not acquired, they will be excluded from the analysis.

### 4.6 Assessment of risk of bias

At least two review investigators (Zhu M and Liu XJ) independently assessed risk of bias of the included RCTs by using the following criteria described in the Cochrane Handbook for Systematic Reviews of Interventions: random sequence generation (selection bias), allocation concealment (selection bias), blinding of participants and personnel (performance bias), blinding of outcome assessment (detection bias), incomplete outcome data (attrition bias), selective reporting (reporting bias), and other bias.^[24,25]^ Evidence quality will be classified as low risk, high risk, or unclear risk of bias. EPOC guidelines will be used to assess the risks of non-RCTs.^[26]^ Any disagreements will be resolved via discussion with a third researcher (Zhou CH).

### 4.7 Data synthesis

We will utilize Review Manager 5.3 (Nordic Cochran Centre, Copenhagen, Denmark) and Stata 14.0 (Stata Corp, College Station, TX) statistical software to pool the data and carry out the data analysis. Heterogeneity between studies will be assessed using the Cochran's Q and Higgins *I*^2^ statistic. *P* < .1 for the Chi^2^ statistic or an *I*^2^ > 50% will be considered as showing considerable heterogeneity.^[27]^ A fixed effect model will be used to calculate the outcomes when statistical heterogeneity is absent; otherwise, the random effects model was considered according to the DerSimonian and Laird method.^[28]^ Continuous data will be presented as standardized mean difference (SMD) with their confidence intervals (CIs). Dichotomous data will be recorded as risk ratio (RR) with 95% CIs. For survival outcomes, hazard ratios (HRs) with corresponding 95% CIs will be extracted from trials or be estimated from Kaplan–Meier survival curves by established methods.^[29]^ A two-tailed *P* < .05 was considered statistically significant.

### 4.8 Subgroup and meta-regression analysis

Subgroup and meta-regression analysis will be conducted to explore the source of heterogeneity with respect to tumor stage, region, course of treatment and therapeutic regimens, et al.

### 4.9 Sensitivity analysis

Sensitivity analysis will be carried out to assess the reliability and robustness of the pooled results via eliminating trials with low quality. A summary table will report the results of the sensitivity analyses.

### 4.10 Other relevant information

#### 4.10.1 Publication bias analysis

Funnel plot, Begg's and Egger regression test will be performed to analyze the existence of publication bias if 10 or more studies are included in the meta-analysis.^[30–32]^ If reporting bias is suspected, we will consult the study author to get more information. If publication bias existed, a trim-and-fill method should be applied to coordinate the estimates from unpublished studies, and the adjusted results were compared with the original pooled RR.^[33,34]^

#### 4.10.2 Evidence evaluation

The evidence grade will be determined by using the guidelines of the Grading of Recommendations, Assessment, Development, and Evaluation (GRADE). The quality of all evidence will be evaluated as 4 levels (high, moderate, low, and very low).^[24,35]^

### 4.11 Dissemination plans

We will disseminate the results of this systematic review by publishing the manuscript in a peer-reviewed journal.

## 5 Discussion

The chemoradiotherapy regimens commonly used to treat HCC often cause serious adverse effects, which severely affect the immune function and QoL of HCC patients.^[11]^ Therefore, seeking an alternative therapy that can improve the immune function and QoL of patients is urgently required for tumor treatment. SC/VB6 is a famous insect-derived traditional Chinese medicine preparation that manufactured by Guizhou Baiqiang and Shanghai new pioneer Pharmaceutical Co., Ltd. It have been approved by Chinese State Food and Drug Administration (SFDA), and granted the Manufacturing Approve Number accordingly (H20053863 and H20045312). Several studies have confirmed that SC/VB6 plays a unique role in improving host immunity, decreasing cancer relapses and lowering the toxic effects of chemotherapy.^[11,16,22,36–38]^

### 5.1 Strengths and limitations

Even though there was statistical analysis of published clinical trials, the exact effects of SC/VB6 injection on survival, liver function, immune function, and QoL in patients with HCC were still not systematically investigated. This systematic review will conduct a systematic, comprehensive and objective evaluation of SC/VB6-based adjuvant therapy. The findings of this analysis will provide a helpful evidence for clinicians to formulate the best postoperative adjuvant treatment strategy for patients with advanced HCC, and also provide scientific clues for researchers in this field. There may be a language bias with the limitation of English and Chinese studies.

## Author contributions

**Conceptualization:** Min Zhu, Juan Li.

**Data curation:** Min Zhu, Xiujing Liu.

**Formal analysis:** Min Zhu, Xiujing Liu.

**Funding acquisition:** Changhui Zhou.

**Investigation:** Min Zhu, Xiujing Liu, Changhui Zhou.

**Methodology:** Min Zhu, Xiujing Liu, Changhui Zhou.

**Project administration:** Juan Li.

**Resources:** Min Zhu, Juan Li.

**Software:** Min Zhu, Juan Li.

**Supervision:** Min Zhu, Juan Li.

**Validation:** Changhui Zhou, Juan Li.

**Visualization:** Min Zhu, Xiujing Liu.

**Writing – original draft:** Min Zhu, Xiujing Liu, Changhui Zhou.

**Writing – review & editing:** Changhui Zhou, Juan Li.
